# Gut *Lactobacillus* Level Is a Predictive Marker for Coronary Atherosclerotic Lesions Progress and Prognosis in Patients With Acute Coronary Syndrome

**DOI:** 10.3389/fcimb.2021.687827

**Published:** 2021-09-07

**Authors:** Jing Gao, Jie Wang, Li-Li Zhao, Ting-Ting Yao, Yang Chen, Jing Ma, Xu Zhang, Jing-Xian Wang, Yuan Wang, Zhuang Cui, Yin Liu

**Affiliations:** ^1^Thoracic Clinical College, Tianjin Medical University, Tianjin, China; ^2^Chest Hospital, Tianjin University, Tianjin, China; ^3^Cardiovascular Institute, Tianjin Chest Hospital, Tianjin, China; ^4^School of Public Health, Tianjin Medical University, Tianjin, China; ^5^Department of Cardiology, Tianjin Chest Hospital, Tianjin, China

**Keywords:** acute coronary syndrome, gut microbiota, coronary atherosclerotic lesion, myocardial necrosis, prognosis

## Abstract

**Background:**

Gut microbiota dysbiosis can contribute to the progression of atherosclerosis. We investigated the association of the gut microbiota and the severity of coronary artery lesions and prognosis of patients with ACS.

**Methods:**

In this case-control study, 402 ACS patients and 100 controls were enrolled from June 2017 to December 2018. The number of bacterial species was determined by real-time PCR. A SYNTAX score was calculated for all ACS patients based on their coronary angiography results.

**Results:**

Compared with the healthy controls, the gut microbial levels in *Escherichia coli*, *Streptococcus*, and *Enterobacteriaceae* were significantly increased in ACS patients, while the *Lactobacillus* level was significantly decreased. *Lactobacillus* level was as an independent predictor of disease severity on the coronary angiography [high vs. low SYNTAX score: adjusted odds ratio (aOR) = 0.024, 95% confidence interval (CI): 0.004–0.155] and myocardial necrosis [high vs. low cardiac troponin T (cTNT): aOR = 0.317, 95% CI: 0.099–0.914]. Subsequently, a higher *Lactobacillus* level was associated with a lower risk of an all-cause death [adjusted hazard ratio (aHR) = 0.239; 95% CI: 0.093–0.617] and major adverse cardiac events (MACE) in ACS patients (aHR = 0.208; 95% CI: 0.081–0.531). After stratifying by the type of ACS, a higher *Lactobacillus* level was significantly associated with the decreased risks of high SYNTAX score, all-cause death, and MACE in the STEMI subgroup but not in the NSTEMI and UAP subgroups.

**Conclusions:**

Lower *Lactobacillus* levels may indicate a higher risk of a more severe coronary atherosclerotic lesions and myocardial necrosis and worse prognosis for patients with ACS, particularly in the STEMI subgroup.

## Introduction

Cardiovascular disease (CVD) is one of the leading causes of death globally, and places a large economic burden on healthcare systems worldwide (Liu et al., 2018). Despite the management of the traditional risk factors, such as hypertension, dyslipidemia, smoking, and diabetes, and the use of modern pharmacotherapies, including high potency statin therapy, at least a 50% residual risk of CVD remains in many patients (Ridker et al., 2008; Writing Group et al., 2016). Therefore, there is an interest in identifying novel CVD risk factors to improve our understanding of the processes that contribute to pathogenesis of CVD, and the prevention and treatment of CVD (Wang, 2008).

Recent studies have found that variations in the gut microbiota can promote atherosclerosis through a variety of metabolic pathways, oxidative stress, and inflammatory response (Wang et al., 2011; Jandhyala et al., 2015; Korem et al., 2015; Tang and Hazen, 2017; Yissachar et al., 2017). An imbalance of the gut microecology leads to a disorder of the flora which can interfere with the basic metabolic processes of the host and lead to the development of CVD such as coronary artery disease (CAD), hypertension, and heart failure (Tang et al., 2015; Senthong et al., 2016b; Li J. et al., 2017; Santisteban et al., 2017).

The SYNTAX [SYNergy between percutaneous coronary intervention with (paclitaxel-eluting) TAXUS stent and cardiac surgery] trial produced the SYNTAX score, which is an angiographic scoring system that indicates the complexity and burden of atherosclerotic CAD (Sianos et al., 2005; Ikeda et al., 2012). According to the 2018 European Society of Cardiology/European Association for Cardio-Thoracic Surgery (ESC/EACTS) guidelines on myocardial revascularization (Neumann et al., 2019), the SYNTAX score can be used to evaluate the severity of coronary stenosis. The anatomical SYNTAX score has been shown to predict the occurrence of major adverse cardiac events (MACE) and the long-term prognosis of stable patients with CAD who have undergone coronary revascularization (Serruys et al., 2009; Mohr et al., 2013).

In our previous study (Gao et al., 2020) of patients with ACS, we found that the levels of *Bifidobacterium*, *Lactobacillus*, *Escherichia coli*, *Streptococcus*, *Helicobacter pylori*, and *Enterobacteriaceae* in the gut microbiota were significantly different between the ACS patients and healthy controls. In addition, we found that ACS and incident post-ST-segment elevation myocardial infarction (post-STEMI) MACE may be associated with the gut bacteria choline metabolite trimethylamine N-oxide (TMAO) (Gao et al., 2020). However, we did not determine whether gut microbe-related ACS and TMAO-related post-STEMI MACE were associated with the severity of the coronary artery lesions.

Thus, in the current study, we investigated if the gut microbiota was associated with the severity of the coronary artery lesions and post-percutaneous coronary intervention (PCI) MACE in patients with ACS.

## Materials and Methods

### Study Design

This study enrolled 402 ACS patients, including 272 with ST-segment elevation myocardial infarction (STEMI), 75 with non-ST-segment elevation myocardial infarction (NSTEMI), and 55 with unstable angina (UA) treated in the cardiac/coronary care unit (CCU) of Tianjin Chest Hospital from June 2017 to December 2018. In addition, 100 healthy subjects who received routine examinations at the hospital health examination center of Tianjin Chest Hospital during the same period were included as control subjects. The routine examinations included echocardiography and coronary computed tomography angiography (CTA). All control subjects had a negative CTA image (defined as no single coronary segment with stenosis).

The study protocol was approved by the Ethics Committee of Tianjin Chest Hospital (No. 2018KY-010-01), and all subjects provided a signed informed consent to participate. All procedures performed were in accordance with the ethical standards of the Helsinki Declaration and its later amendments, or comparable ethical standards.

### Diagnostic Criteria for Acute Coronary Syndrome

1. STEMI: a) Cardiac troponin (cTn) I/T > the upper limit of the normal reference value, or creatine kinase (CK) isoenzyme > the upper limit of the normal reference value; b) electrocardiogram (ECG) showing an ST-segment elevation on two or more adjacent leads; and c) one or more of the following: persistent ischemic chest pain, abnormal segmental wall motion on ECG, and abnormal coronary angiography.
2. NSTEMI: a) Cardiac troponin and/or CK-MB were above the reference limit of 99%; b) ECG showing an ST-segment depression or T-wave inversion and/or persistent chest pain for more than 30 minutes.
3. UA: a) cTnI/T negative; b) ischemic chest pain; and c) ECG showing transient ST-segment depression/low-level T-wave or inverted, rare ST-segment elevation.

All ACS patients received coronary angiography, had one or more coronary artery stenosis (stenosis ≥ 50%), and received percutaneous coronary intervention (PCI) within 12 hours or 72 hours.

Participants were excluded from the study if they have any of the following criteria: 1) History of organic digestive system disease or digestive tract surgery; 2) History of stroke, kidney disease, or respiratory diseases; 3) History of smoking or alcohol abuse; or 4) Infection within 1 month of the study or the use of a probiotic, antacid, or antibiotic.

Data were collected from the electronic medical record system. Information collected included age, sex, height, weight, history of past illnesses, blood pressure, heart rate, blood biochemical indexes, ECG results, and coronary angiography results.

### Specimens

Fasting blood specimens and fresh stool specimens were collected during the first day of admission. Blood specimens were centrifuged at 3,000 rpm for 10 minutes at 4°C, and the supernatant was frozen and stored at -80°C until use. A first morning fresh fecal specimen (>300 mg) was collected from all subjects in a specimen container, sealed, and transported to the laboratory at 4°C. Fecal specimens (300 mg) were placed into a sterile, externally-circulated cryotube that was sealed and stored at -156°C.

### DNA Extraction and 16S rDNA Gene Sequencing

Genomic DNA was extracted from the specimens using the cetyltrimethylammonium bromide method. Genomic DNA was diluted to 1 ng/μl and used as a template, and the 16 S V4 region was the amplified target. The primer sequences were 515 Forward (5′-GTGCCAGCMGCCGCGGTAA-3′) and 806 Reverse (5′-GGACT CHVGGGTWTCTAAT-3′). Polymerase chain reaction (PCR) was performed using the Phusion High-Fidelity PCR Master Mix. The PCR products were purified using a Thermo Scientific kit (Thermo Fisher Scientific, Waltham, MA, USA). The bacterial 16S rDNA sequences of 60 ACS patients and 30 healthy controls were generated to analyze the specific gut microbial taxa associated with ACS, as shown in our prior study (Gao et al., 2020), indicating that the abundances of 18 gut microbial families or genera were significantly different between the ACS and control groups. Thus, by real-time quantitative PCR in 402 ACS and 100 control subjects, the present study further determined whether the top six microbes (*Bifidobacteria*, *Lactobacillus*, *Escherichia coli*, *Streptococcus*, *Helicobacter pylori*, and *Enterobacteriaceae*) selected from 18 gut microbial families or genera are associated with the severity of coronary lesions, MACE, and all-cause death.

### Real-Time Quantitative PCR

SYBR Green II real-time quantitative PCR was used to detect the changes of two intestinal probiotic bacteria (*Bifidobacteria*, *Lactobacillus*) and four intestinal pathogens (*E. coli*, *Streptococcus*, *H. pylori*, and *Enterobacteriaceae*) in the fecal samples. PCR was performed after fecal bacterial DNA was extracted from the stool specimens. The reaction primer of the 16S rRNA of the appropriate reference organisms was designed by reference to the literature (Nadkarni et al., 2002; Sianos et al., 2005; Serruys et al., 2009; Ikeda et al., 2012; Mohr et al., 2013; Tang et al., 2015; Senthong et al., 2016b; Neumann et al., 2019), and was synthesized by Thermo Fisher Scientific Co., Ltd. The primer sequences are shown in **Supplementary Table 1**.

The reaction was carried out using the TB Green ™ Premier Ex Taq ™ II (Tli RNaseH Plus) (Takara, Shiga, Japan). The reaction system was comprised of the Real Master Mix (10 μl), upstream and downstream primers (0.8 μl), ROX II (0.4 μl), template DNA (2 μl), and DEPC water (6 μl) for a total reaction volume of 20 μl. The reaction conditions were as follows: pre-denaturation at 95°C for 5 min, denaturation at 95°C for 15 s (*Bifidobacteria*, 58°C; *Lactobacillus*, 58°C; *E. coli*, 60°C; *Streptococcus*, 60°C; *H. pylori*, 62°C; *Enterobacteriaceae*, 63°C), annealing for 34 s, extension for 60 s at 72°C, and circulation for 40 times.

Standard product calibration and negative control were used for each reaction, and the specificity of the PCR products were analyzed according to the dissolution curve after the completion of the reaction. Based on the read fluorescence data, the System SDS Interface software (7500) automatically analyzes the Ct value and generates a standard curve. Bacterial quantity was expressed as log^10^ bacteria per gram of stool.

### Quantification of Bacteria in Fecal Specimens

The amplification curve indicates the relation between the number of template cycles and fluorescence intensity of 1 × 10^2^ to 1 × 10^7^ copies, and can be obtained by real-time quantitative PCR with a dilution of 10 times series of standard products (**Supplementary Figure 1**). It can be seen from the diagram (**Supplementary Figure 1**) that the fluorescent intensity of the templates with different copy numbers increases with the increase of cycle number, and the curve tends to be parallel after an exponential amplification period; that is to say the “platform effect” appears. The corresponding relation between the copy number of the template during the exponential amplification period and the fluorescent accumulation value forms the basis for quantification. The standard curve for each bacteria was obtained by using the logarithm of the standard product with a different copy number as the transverse coordinate, and the initial cycle number (Ct) which reached the fluorescent threshold during the PCR reaction as the longitudinal coordinate, which thus provided the reference standard for the quantification of the sample to be tested (**Supplementary Figure 2**). After each quantitative PCR reaction, disassociation curve analysis was performed. As shown in **Supplementary Figure 3**, the melting curves are all single peaks indicating that the amplification product is single, and the target DNA fragment bound to the SYBR Green fluorescent dye avoids the false positive results during the quantitative PCR detection.

The copy numbers of the different bacteria tested for can be obtained by comparing the Ct value with the standard curve of the standard bacterial strain. The quantitative results are produced directly by the software of the real-time quantitative PCR instrument.

### SYNergy Between Percutaneous Coronary Intervention With (Paclitaxel-Eluting) TAXUS Stent and Cardiac Surgery Score and Patient Follow-Up

All ACS patients had a SYNTAX score calculated based on their coronary angiography results, and were followed-up for at least 18 months. SYNTAX is an anatomically-based scoring system that quantitatively characterizes the coronary vessels according to the number, complexity, location, and functional aspects of the obstructive lesions identified on coronary angiography (Kim and Park, 2018). Vessels with a diameter > 1.5 mm that contain lesions with ≥50% stenosis were assessed. Each coronary segment is given a weight factor that is determined by the lesion location and severity. Lesion characteristics, including total occlusion, trifurcation, bifurcation, calcification, tortuosity, length > 20 mm, thrombus, and diffuse or small-vessel disease, are combined to give a final score *via* the SYNTAX calculator. SYNTAX score was grouped into low SYNTAX, medium SYNTAX, and high SYNTAX according to previous studies (Serruys et al., 2009; Farooq et al., 2014; Sousa-Uva et al., 2019; Thuijs et al., 2019). Patients were followed-up at 6, 12, 24, and 36 months after diagnosis using telephone and/or interview after the initial appointment by trained nurses or cardiologists. The occurrence of a first MACE was regarded as the follow-up endpoint. MACE included cardiac death, non-fatal ischemic stroke, recurrent MI, need for emergency or repeat revascularization, and re-hospitalization for heart failure.

### Statistical Analysis

Quantitative data were imported into the SPSS version 23.0 statistical software for analysis, and the measurement data were expressed in quartiles. The independent sample t-test or nonparametric Wilcoxon rank-sum test were used for intergroup comparisons. Logistic regression was used to analyze the factors influencing the onset of symptoms, coronary artery disease severity, and prognosis of ACS patients. Multivariate Cox regression was performed to determine predictors for the primary endpoints using univariate analysis parameters with a p < 0.05. Receiver operating characteristic (ROC) curve analysis was performed to identify 1-year MACE and all-cause mortality rates with respect to the identified prognostic markers. The Kaplan-Meier method was used to compare the MACE-occurring survival time and all-cause death-occurring survival time for patients with high and low *Lactobacillus* levels, and curves were compared using the log-rank test. Values of p < 0.05 were considered to indicate a statistical significance.

## Results

### Patient Characteristics

**Figure 1:**
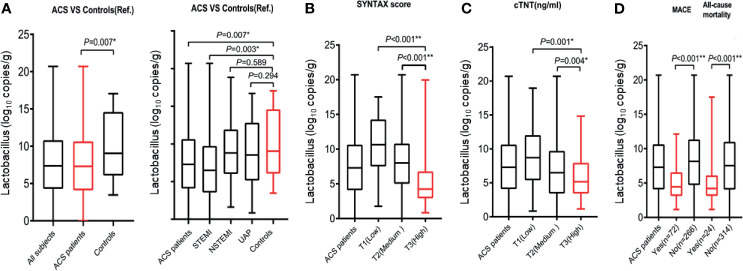
Relationship between Lactobacillus and measures of coronary artery lesion and clinical myonecrosis. **(A)** Left panel, Lactobacillus levels significantly increased in the ACS patients compared with that in the control group. Right panel, Lactobacillus levels significantly increased in the STEMI subgroup compared with that in the control group. In the 402 ACS patients with an evidence of coronary artery lesion, Lactobacillus levels were significantly higher with **(B)** increasing SYNTAX score, **(C)** clinical myonecrosis [quantified by cardiac troponin T (cTnT)] tertiles, and **(D)** proportion of all-cause death and MACE. *p < 0.05; **p < 0.001.

The baseline characteristics of the 402 ACS patients and 100 healthy control patients are shown in **Table 1**. In the ACS group, the median patient age was 64 years, 70% were men, 65.9% had hypertension, and 27.3% had diabetes. In the control group, the median patient age was 64 years, 61% were men, 41.0% had hypertension, and 10.0% had diabetes. The amounts of *Escherichia coli*, *Streptococcus*, and *Enterobacteriaceae* were significantly greater in the ACS group than in the control group (p < 0.05, **Table 1**). On the other hand, the amount of *Lactobacillus* was significantly lower in the ACS group than in the control group (p < 0.05, **Figure 1A** and **Table 1**). Compared to the control group, ACS patients had a lower *Lactobacillus level*, a higher *Enterobacteriaceae* level, and a higher proportion of hypertension or diabetes (all p < 0.05, **Table 1**). After stratifying by the type of ACS (STEMI, NSTEMI, and UAP), the *Lactobacillus* level significantly increased in the ACS patients and STEMI subgroup compared with that in the control group (**Figure 1A**).

**Table 1:** Patient characteristics.

Characteristics	ACS (n = 402)	CTR (n = 100)	*p*-value^a^
Ages (years)	64.00 (56.00,73.00)	64.00 (59.50,67.50)	0.408
Male, n (%)	256 (63.70%)	61 (61.00%)	0.732
BMI (kg/m^2^)	25.53 (24.14,26.77)	22.72 (20.30,25.15)	<0.001*
History of past illness			
Hypertension, n (%)	265 (65.90%)	41 (41.00%)	0.002*
Diabetes, n (%)	121 (27.30%)	10 (10.00%)	0.006*
Admission to hospital			
Systolic pressure (mmHg)	130.00 (120.00,147.25)	120.00 (103.00,126.5)	<0.001*
Heart rate (beats per min)	70.00 (65.00,80.00)	74.00 (67.50,77.00)	0.485
LVEF (%)	53.00 (45.00,58.70)	65.00 (63.00,67.00)	<0.001*
Laboratory index			
cTNT max (ng/mL)	1.03 (0.02,4.35)	–	–
BNP max (pg/mL)	708.90 (157.25,1857.00)	–	–
Lp (a) (nmol/L)	35.60 (13.15,78.88)	17.60 (6.90,55.60)	0.017*
TG (mmol/L)	1.45 (1.12,2.02)	1.01 (0.80,1.66)	0.056
TC (mmol/L)	4.36 (3.72,5.05)	4.63 (3.83,5.36)	0.183
LDL (mmol/L)	2.92 (2.23,3.51)	2.94 (2.25,3.59)	0.780
HDL (mmol/L)	1.04 (0.85,1.25)	1.34 (1.10,1.60)	<0.001*
APOB (g/L)	1.03 (0.83,1.22)	0.89 (0.70,1.08)	0.007*
UA (mmol/L)	322.00 (259.75,383.25)	316.00 (254.50,361.50)	0.432
*Bifidobacteria* (log_10_ copies/g)	4.80 (3.53,9.40)	5.10 (3.67,9.72)	0.302
*Lactobacillus* (log_10_ copies/g)	7.32 (4.21,10.27)	9.04 (6.07,14.59)	0.007*
*Escherichia coli* (log_10_ copies/g)	8.43 (5.81,11.49)	5.80 (3.90,8.06)	<0.001*
*Streptococcus* (log_10_ copies/g)	9.46 (6.69,11.70)	7.74 (6.24,9.39)	0.018*
*Helicobacter pylori* (log_10_ copies/g)	2.15 (0.89,3.01)	1.78 (0.84,2.86)	0.941
*Enterobacteriaceae* (log_10_ copies/g)	14.25 (10.97,17.13)	10.97 (7.11,13.36)	<0.001*

ACS, acute coronary syndrome; CTR, control group; BMI, body mass index; LVEF, Left ventricular ejection fraction; cTNT max, cardiac troponin T max; BNP max, B-type natriuretic peptide max; L (a), lipoprotein a; Hcy, homocysteine; Crp, C-reactive protein; TG, triglyceride; TC, total cholesterol; LDL, low density lipoprotein; HDL, high density lipoprotein; APOB, apolipoprotein B; UA, uric acid.

^a^The independent sample t-test or nonparametric Wilcoxon rank-sum test were used for intergroup comparisons. *p < 0.05.

Multivariate logistic regression analysis adjusting for the traditional risk factors, including age, sex, hypertension, diabetes, and LDL-C, HDL-C, and TG levels, showed that a low *Lactobacillus* or a high *Enterobacteriaceae* level increased the risk of ACS (all, p < 0.05, **Supplementary Table 2**); however, hypertension or diabetes did not increase the risk of ACS (all, p > 0.05, **Supplementary Table 2**).

### Relations Between the Gut Microbiota and Coronary Artery Lesions

The median *Lactobacillus* level was 7.32 log_10_ copies/g [interquartile range (IQR): 4.21 to 10.27] in 402 patients with ACS, and 50 (12.4%), 239 (59.5%), and 113 (28.1%) patients had low (SS < 12), medium (12 ≦ SS ≦ 36), and high (SS > 36) SYNTAX scores, respectively (**Table 2**). *Lactobacillus* level significantly decreased as the SYNTAX score (SS) increased in patients with ACS (p < 0.05, **Figure 1B** and **Table 2**). In contrast, age, left ventricular ejection fraction (LVEF), and serum levels of CK, CK-MB, cardiac troponin T (cTNT), and B-type natriuretic peptide (BNP) were significantly positively correlated with the SYNTAX score (all, p < 0.05, **Table 2**). In addition, *Lactobacillus* level also significantly decreased as the cTNT increased in patients with ACS (p < 0.05, **Figure 1C**).

**Table 2:** Demographic, biochemical characteristics, and bacterial quantification of patients grouped according to the SYNTAX score.

Characteristics	All patients (n = 402)	SS<12 (Low) (n = 50)	12≤SS ≤ 36 (Medium) (n = 239)	SS>36 (High) (n = 113)	*p-*value^a^
Ages (years)	64.00 (56.00,73.00)	60 (52.75,79.90)	64.00 (55.25,79.90)	66.00 (60.00,75.75)	0.002*
Male, n (%)	256 (63.70%)	39 (78.00%)	149 (62.10%)	68 (60.70%)	0.077
BMI (kg/m^2^)	25.53 (24.14,26.77)	25.59 (24.11,29.41)	26.77 (24.31,28.50)	25.14 (23.66,28.09)	0.067
**History of past illness**					
Hypertension, n (%)	265 (65.90%)	30 (60.00%)	147 (61.30%)	88 (78.60%)	0.003*
Diabetes, n (%)	121 (30.10%)	11 (22.00%)	77 (32.10%)	33 (29.50%)	0.346
Hyperlipidemia, n (%)	150 (37.30%)	25 (50.00%)	85 (35.40%)	40 (35.70%)	0.140
MI, n (%)	60 (14.90%)	7 (14.00%)	36 (15.00%)	17 (15.20%)	0.980
PCI, n (%)	79 (19.70%)	11 (22.00%)	50 (20.80%)	18 (16.10%)	0.523
**Admission to hospital**					
Systolic pressure (mmHg)	130.00 (120.00,147.25)	132.50 (121.50,147.00)	130.00 (120.00,147.00)	130.00 (118.00,146.00)	0.480
Heart rate (beats per min)	70.00 (65.00,80.00)	70.00 (64.25,78.50)	70.00 (64.00,80.00)	70.00 (65.00, 80.00)	0.733
LVEF, (%)	53.00 (45.00,58.70)	56.00 (46.00,60.00)	55.00 (45.00,59.00)	49.00 (44.00,56.00)	0.003*
**Laboratory index**					
CK max (U/L)	426.00 (91.00,1580.00)	119.00 (84.75,873.00)	491.50 (98.75,1577.50)	510.50 (98.75,1952.75)	0.034*
CK-MB max (U/L)	48.00 (16.00,146.50)	18.00 (13.75,87.25)	53.50 (16.00,137.75)	60.50 (16.25,171.75)	0.002*
cTNT max (ng/mL)	1.03 (0.02,4.35)	0.05 (0.01,2.66)	1.04 (0.02,4.64)	1.36 (0.04,4.44)	0.017*
BNP max (pg/mL)	708.90 (157.25,1857.00)	231.45 (47.38,1.12.50)	746.30 (149.30,4497.20)	890.00 (272.40,1755.00)	0.008*
Lp (a) (nmol/L)	35.60 (13.15,78.88)	46.80 (18.30,100.18)	35.30 (12.60,76.10)	34.20 (12.15,78.85)	0.311
TG (mmol/L)	4.36 (3.72,5.05)	4.59 (3.89,5.17)	4.25 (3.55,5.01)	4.46 (3.81,5.20)	0.472
TC (mmol/L)	1.45 (1.12,2.02)	1.42 (1.05,2.10)	1.47 (1.13,1.97)	1.44 (1.01,2.35)	0.136
LDL (mmol/L)	2.92 (2.23,3.51)	3.05 (2.52,3.30)	2.85 (2.16,3.49)	3.00 (2.42,3.66)	0.197
HDL (mmol/L)	1.04 (0.85,1.25)	1.07 (0.84,1.25)	1.03 (0.85,1.26)	1.03 (0.87,1.25)	0.962
APOB (g/L)	1.03 (0.83,1.22)	1.09 (0.98,1.24)	1.00 (0.81,1.21)	1.07 (0.83,1.26)	0.030*
UA (mmol/L)	322.00 (259.75,383.25)	339.00 (295.00,404.50)	320.00 (262.25,377.50)	320.00 (245.20,376.50)	0.134
***Bifidobacteria*** (log_10_ copies/g)	4.80 (3.53,9.40)	5.21 (3.09,9.58)	4.92 (2.90,7.74)	4.34 (2.15,6.01)	0.528
***Lactobacillus*** (log_10_ copies/g)	7.32 (4.21,10.27)	10.63 (7.50,14.26)	8.01 (5.01,10.79)	4.24 (2.92,6.78)	<0.001*
***Escherichia coli*** (log_10_ copies/g)	8.43 (5.81,11.49)	7.77 (6.03,11.38)	8.83 (6.04,11.67)	8.17 (5.48,11.34)	0.341
***Streptococcus*** (log_10_ copies/g)	9.46 (6.69,11.70)	9.42 (5.32,11.58)	9.63 (6.91,11.81)	9.05 (6.23,11.01)	0.912
***Helicobacter pylori*** (log_10_ copies/g)	2.15 (0.89,3.01)	1.58 (0.71,11.58)	1.77 (0.83,3.01)	1.90 (0.86,2.76)	0.727
***Enterobacteriaceae*** (log_10_ copies/g)	14.25 (10.97,17.13)	13.12 (9.28,16.09)	14.53 (11.45,17.66)	13.93 (10.73,16.67)	0.109

SS, SYNTAX score; BMI, body mass index; MI, myocardial infarction; PCI, percutaneous coronary intervention; LVEF, Left ventricular ejection fraction; CK max, creatine kinase max; CK-MB max, creatine kinase isoenzyme; cTNT max, cardiac troponin T max; BNP max, B-type natriuretic peptide max; L (a), lipoprotein a; Hcy, homocysteine; Crp, C-reactive protein; TG, triglyceride; TC, total cholesterol; LDL, low density lipoprotein; HDL, high density lipoprotein; APOB, apolipoprotein B; UA, uric acid.

^a^Differences in different parameters between the three groups were compared using Kruskal-Wallis nonparametric test. *p < 0.05.

**Figure 2:**
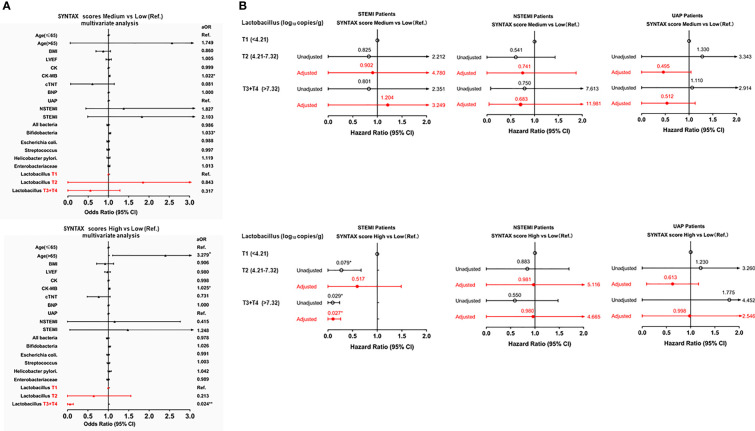
Forest plot to show aORs and 95% CIs for the risks of a high SYNTAX score. **(A)** *upper panel* (ACS patients), medium vs low (reference); *lower panel*, high vs low (reference). **(B)** *upper panel* (STEMI, NSTEMI, and UAP subgroups), medium vs low (reference); *lower panel*, high *vs* low (reference). *p < 0.05; **p < 0.001.

Subsequent multivariate logistic regression analysis adjusting for the traditional risk factors also showed that a higher *Lactobacillus* level (T3+T4, >7.32 log_10_ copies/g) was associated with a decreased risk of severe coronary artery lesions [high vs. low SS: adjusted odds ratio (aOR) = 0.024, 95% confidence interval (CI): 0.004–0.155, p < 0.001, **Figure 2A**], while patients whose ages are above 65 years old were associated with an increased risk of severe coronary artery lesions (high vs. low SS: aOR = 3.279, 95% CI: 1.137–6.480, p < 0.05, **Figure 2A**). However, the type of ACS was not an independent factor for the prediction of the SYNTAX score (p > 0.05, **Figure 2A**). In addition, multivariate logistic regression analysis adjusting for the traditional risk factors showed that a higher *Lactobacillus* level (T3+T4, >7.32 log_10_ copies/g) was associated with a decreased risk of severe myocardial necrosis [high vs. low cTNT: adjusted odds ratio (aOR) = 0.317, 95% CI: 0.099–0.914, p < 0.05, **Supplementary Table 3**].

After stratifying by the type of ACS (STEMI, NSTEMI, and UAP), the results of the univariate logistic regression analysis showed that a higher *Lactobacillus* level was significantly associated with a decreased risk of the high SYNTAX score in the STEMI subgroup but not in the NSTEMI and UAP subgroups (**Figure 2B**).

Next, we analyzed the potential of the stool *Lactobacillus* level as a diagnostic marker of severe coronary artery lesions. ROC analysis revealed that the best diagnostic cutoff value of *Lactobacillus* level was 7.32 log_10_ copies/g, which yielded an area under the curve (AUC) of 0.706, sensitivity of 76%, and specificity of 54% for the diagnosis of severe coronary artery lesions (high SYNTAX score) (**Supplementary Figure 4**).

### Associations of Bacterial Content With Major Adverse Cardiac Event and All-Cause Mortality

A total of 64 ACS patients were lost to follow-up, including 9 cases of patients or family members who refused to provide patient information, 40 cases of mobile phone numbers of patients being empty or wrong, and 15 patients who refused to answer the phone. We excluded these cases from the analysis because we did not get the information of all-cause death and MACE. In addition, we compared the baseline characteristics of 64 (non-follow-up) and 338 (follow-up) ACS patients. The results exhibited that the baseline characteristics of the patients were not significantly different between the two groups (**Tables 3** and **4**). Of the 338 ACS patients, 24 (7.10%) and 72 (21.30%) of the patients had an occurrence of all-cause death and MACE at the 18-month follow-up, respectively (**Tables 3** and **4**). The *Lactobacillus* level, being male, and LVEF were negatively associated with the occurrence of an all-cause death, while age, history of MI, systolic blood pressure, heart rate, BNP level, and UA were positively associated with the occurrence of an all-cause death (all, p < 0.05, **Table 3** and **Figure 1D**). Moreover, the *Lactobacillus* level and LVEF were negatively associated with the occurrence of MACE, while age, history of MI, PCI, and, cTNT, BNP, homocysteine (Hcy), and C-reactive protein (CRP) levels were positively associated with the occurrence of MACE (all, p < 0.05, **Table 4** and **Figure 1D**).

**Table 3:** Baseline data for all-cause death analysis .

Characteristics	All patients (n = 402)	Lost to follow-up (n = 64)	*p-*value^a^	All-cause death at 1 year			*p-*value^a^
				Yes (n = 24)	No (n = 314)		
Ages (years)	64.00 (55.00,72.25)	64.00 (53.00,73.00)	0.804	75.00 (67.25,79.25)		63.00 (55.00,71.25)	<0.001*
Male, n (%)	218 (64.5%)	256 (63.70%)	0.435	10 (41.70%)		208 (66.20%)	0.015*
BMI (kg/m^2^)	25.55 (24.12,26.74)	25.53 (24.14,26.77)	0.563	25.31 (23.70,26.79)		25.56 (24.14,26.71)	0.665
**History of past illness**							
Hypertension, n (%)	223 (66.00%)	265 (65.90%)	0.957	14 (58.30%)		209 (66.60%)	0.412
Diabetes, n (%)	103 (30.50%)	121 (30.10%)	0.708	9 (37.50%)		94 (29.90%)	0.438
Hyperlipidemia, n (%)	131 (38.80%)	150 (37.30%)	0.169	6 (25.00%)		125 (39.80%)	0.151
MI, n (%)	51 (15.10%)	60 (14.90%)	0.833	10 (41.70%)		41 (13.10%)	<0.001*
PCI, n (%)	68 (20.10%)	79 (19.70%)	0.589	8 (33.30%)		60 (19.10%)	0.094
**Admission to hospital**							
Systolic pressure (mmHg)	130.00 (120.00,150.00)	130.00 (120.00,147.25)	0.553	120.00 (111.00,131.50)		132.00 (120.00,150.00)	0.029*
Heart rate (beats per min)	70.00 (64.00,80.00)	70.00 (65.00,80.00)	0.933	75.00 (70.00,84.75)		70.00 (64.00,80.00)	0.033*
LVEF (%)	52.50 (45.00,59.00)	53.00 (45.00,58.70)	0.987	42.00 (31.25,50.00)		54.00 (45.00,59.00)	<0.001*
**Laboratory index**							
CK max (U/L)	440.00 (94.00,1580.00)	426.00 (91.00,1580.00)	0.740	857.50 (118.50,2424.25)		418.50 (89.75,1557.50)	0.230
CK-MB max (U/L)	48.50 (16.00,148.75)	48.00 (16.00,146.50)	0.407	107.00 (18.25,264.75)		46.50 (16.00,136.75)	0.109
cTNT max (ng/mL)	1.05 (0.02,4.24)	1.03 (0.02,4.35)	0.805	2.09 (0.14,10.00)		0.98 (0.02,3.93)	0.05044
BNP max (pg/mL)	704.45 (155.08,1805.25)	708.90 (157.25,1857.00)	0.776	3674.50 (1315.25,19322.00)		638.10 (126.35,1523.00)	<0.001*
Hcy (umol/L)	13.80 (11.20,18.85)	13.8 (11.10,18.85)	0.789	16.65 (12.23,22.70)		13.65 (11.18,18.48)	0.063
Crp (ng/ml)	4.26 (1.55,11.23)	4.30 (1.55,12.40)	0.706	12.57 (3.16,34.14)		4.03 (1.48,10.57)	0.017*
Lp (a) (mg/L)	35.60 (13.15,77.38)	33.15 (12.53,91.25)	0.827	35.60 (13.58,76.80)		33.15 (8.10,120.03)	0.981
TC (mmol/L)	1.50 (1.13,2.03)	35.60 (13.15,78.88)	0.827	1.41 (1.11,2.68)		1.52 (1.13,5.08)	0.110
TG (mmol/L)	4.35 (3.40,5.06)	4.36 (3.72,5.05)	0.927	4.26 (3.10,4.84)		4.37 (3.76,5.08)	0.360
LDL (mmol/L)	2.92 (2.23,3.52)	1.45 (1.12,2.02)	0.187	2.78 (1.83,3.68)		2.93 (2.23,3.52)	0.575
HDL (mmol/L)	1.03 (0.85,1.25)	2.92 (2.23,3.51)	0.749	1.01 (0.85,1.22)		1.03 (0.85,1.25)	0.927
APOB (g/L)	1.03 (0.84,1.22)	1.000 (0.82,1.23)	1.000	1.04 (0.85,1.22)		0.96 (0.76,1.23)	0.400
UA (mmol/L)	322.00 (259.75,383.25)	299.00 (252.25,376.00)	0.237	397.50 (285.50,484.75)		322.00 (260.00,376.00)	0.015*
***Bifidobacteria* (log_10_ copies/g)**	4.80 (3.53,9.40)	4.78 (3.86,8.56)	0.185	4.62 (3.98,6.65)		4.85 (3.75,8.57)	0.642
***Lactobacillus***	7.32 (4.21,10.27)	7.25 (3.94,10.81)	0.612	4.21 (3.13,6.11)		7.54 (4.04,11.00)	<0.001*
***Escherichia coli***	8.43 (5.81,11.49)	8.29 (5.67,11.34)	0.181	8.55 (6.48,10.92)		8.23 (5.65,11.35)	0.547
***Streptococcus***	9.46 (6.69,11.70)	9.44 (6.51,11.66)	0.172	10.04 (7.52,12.19)		9.36 (6.37,11.46)	0.226
***Helicobacter pylori***	2.15 (0.89,3.01)	1.78 (0.83,2.91)	0.894	1.80 (0.91,2.10)		1.78 (0.83,3.06)	0.516
***Enterobacteriaceae***	14.25 (10.97,17.13)	13.99 (10.91,17.01)	0.215	15.80 (12.68,18.14)		13.96 (10.77,17.01)	0.226

BMI, body mass index; MI, myocardial infarction; PCI, percutaneous coronary intervention; LVEF, Left ventricular ejection fraction; CK max, creatine kinase max; CK-MB max, creatine kinase isoenzyme; cTNT max, cardiac troponin T max; BNP max, B-type natriuretic peptide max; Hcy, homocysteine; Crp, C-reactive protein; L (a), lipoprotein a; TG, triglyceride; TC, total cholesterol; LDL, low density lipoprotein; HDL, high density lipoprotein; APOB, apolipoprotein B; UA, uric acid.

^a^The independent sample t-test or nonparametric Wilcoxon rank-sum test were used for intergroup comparisons. *p < 0.05.

**Table 4:** Baseline data for major adverse cardiovascular events analysis.

Characteristics	All patients (n = 402)	Lost to follow-up (n = 64)	*p-*value^a^	MACE at 1 year		*p-*value^a^
				Yes (n = 72)	No (n = 266)	
Ages, (years)	64.00 (53.00,73.00)	64.00 (57.25,73.00)	0.804	63.00 (55.00,71.00)	70.00 (61.00,79.00)	<0.001*
Male, n (%)	256 (63.70%)	38 (59.40%)	0.435	33 (58.90%)	185 (65.60%)	0.340
BMI, kg/m2	25.53 (24.14,26.77)	25.37 (23.58,26.76)	0.563	25.21 (23.45,26.37)	25.58 (24.22,26.78)	0.110
**History of past illness**						
Hypertension, n (%)	265 (65.90%)	42 (65.60%)	0.957	37 (66.10%)	186 (66.00%)	0.987
Diabetes, n (%)	121 (30.10%)	18 (28.10%)	0.708	17 (30.40%)	86 (30.50%)	0.983
Hyperlipidemia, n (%)	150 (37.30%)	19 (29.70%)	0.169	19 (33.90%)	112 (39.70%)	0.417
MI, n (%)	60 (14.90%)	9 (14.10%)	0.833	17 (30.40%)	34 (12.10%)	<0.001*
PCI, n (%)	79 (19.70%)	11 (17.20%)	0.589	17 (30.40%)	51 (18.1%)	0.036*
**Admission to hospital**						
Systolic pressure (mmHg)	130.00 (120.00,147.25)	130.00 (116.50,143.75)	0.553	130.00 (115.50,139.50)	131.00 (120.00,150.00)	0.111
Heart rate (beats per min)	70.00 (65.00,80.00)	70.00 (65.00,78.75)	0.933	72.00 (66.00,80.00)	70.00 (64.00,80.00)	0.401
LVEF (%)	53.00 (45.00,58.70)	53.50 (45.00,58.00)	0.987	47.50 (40.25,56.00)	54.00 (45.00,59.00)	0.002*
**Laboratory index**						
CK max (U/L)	426.00 (91.00,1580.00)	299.00 (88.75,1544.00)	0.740	777.50 (120.50,2087.50)	391.50 (87.25,1557.50)	0.061
CK-MB max (U/L)	48.00 (16.00,146.50)	43.00 (15.00,140.00)	0.407	85.00 (19.25,211.75)	43.00 (16.00,133.00)	0.054
cTNT max (ng/mL)	1.03 (0.02,4.35)	0.90 (0.02,5.71)	0.805	2.11 (0.22,7.80)	0.84 (0.02,3.84)	0.009*
BNP max (pg/mL)	708.90 (157.25,1857.00)	778.65 (152.95,2361.25)	0.776	1725.00 (847.00,4610.00)	534.90 (155.60,1453.50)	<0.001*
Hcy (umol/L)	13.8 (11.10,18.85)	13.75 (10.80,18.98)	0.789	16.15 (11.85,21.63)	13.50 (11.10,18.30)	0.036*
CRP (ng/ml)	4.30 (1.55,12.40)	4.87 (1.45,14.27)	0.706	6.78 (2.60,25.13)	4.02 (1.43,10.20)	0.033*
Lp (a) (mg/L)	35.60 (13.15,77.38)	33.15 (12.53,91.25)	0.827	35.30 (12.60,74.95)	35.70 (13.88,78.35)	0.608
TC (mmol/L)	35.60 (13.15,78.88)	33.15 (12.53,91.25)	0.827	1.41 (1.02,1.88)	1.51 (1.14,2.10)	0.165
TG (mmol/L)	4.36 (3.72,5.05)	4.37 (3.77,5.04)	0.927	4.22 (3.46,4.91)	4.39 (3.77,5.10)	0.192
LDL (mmol/L)	1.45 (1.12,2.02)	1.29 (1.04,1.83)	0.187	2.82 (2.07,3.39)	2.94 (2.24,3.55)	0.385
HDL (mmol/L)	2.92 (2.23,3.51)	2.90 (2.26,3.49)	0.749	1.03 (0.84,1.25)	1.03 (0.85,1.25)	0.646
APOB (g/L)	1.03 (0.84,1.22)	1.000 (0.82,1.23)	1.000	1.02 (0.79,1.15)	1.04 (0.85,1.24)	0.233
UA (mmol/L)	322.00 (259.75,383.25)	299.00 (252.25,376.00)	0.237	344.50 (278.00,433.75)	322.00 (260.00,375.00)	0.066
***Bifidobacteria* (log_10_ copies/g)**	4.80 (3.53,9.40)	4.78 (3.86,8.56)	0.185	4.53 (3.19,6.56)	4.94 (3.31,9.02)	0.581
***Lactobacillus***	7.32 (4.21,10.27)	7.25 (3.94,10.81)	0.612	4.45 (3.13,6.56)	8.16 (4.70,11.34)	<0.001*
***Escherichia coli***	8.43 (5.81,11.49)	8.29 (5.67,11.34)	0.181	8.12 (5.36,10.35)	8.35 (5.90,11.68)	0.244
***Streptococcus***	9.46 (6.69,11.70)	9.44 (6.51,11.66)	0.172	9.65 (6.80,11.92)	9.20 (6.26,11.54)	0.242
***Helicobacter pylori***	2.15 (0.89,3.01)	1.78 (0.83,2.91)	0.894	1.91 (0.89,3.15)	1.69 (0.83,2.87)	0.698
***Enterobacteriaceae***	14.25 (10.97,17.13)	13.99 (10.91,17.01)	0.215	13.96 (11.54,16.20)	14.19 (10.83,17.41)	0.342

MACE, major adverse cardiovascular event; BMI, body mass index; MI, myocardial infarction; PCI, percutaneous coronary intervention; LVEF, Left ventricular ejection fraction; CK max, creatine kinase max; CK-MB max, creatine kinase isoenzyme; cTNT max, cardiac troponin T max; BNP max, B-type natriuretic peptide max; Hcy, homocysteine; Crp, C-reactive protein; L (a), lipoprotein a; TG, triglyceride; TC, total cholesterol; LDL, low density lipoprotein; HDL, high density lipoprotein; APOB, apolipoprotein B; UA, uric acid.

^a^The independent sample t-test or nonparametric Wilcoxon rank-sum test were used for intergroup comparisons. *p < 0.05.

**Figure 3:**
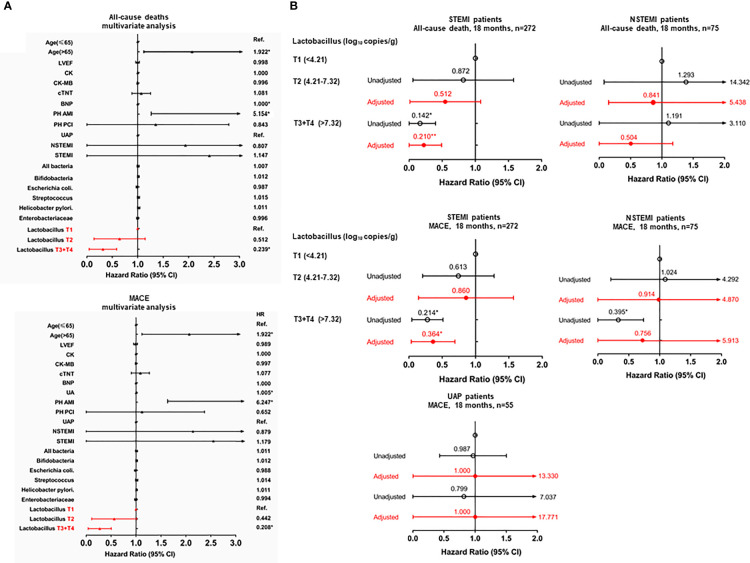
Forest plot to show aHRs and 95% CIs for the all-cause mortality or MACE. **(A)** *upper panel*, 18-months all-cause mortality in ACS patients; *lower panel*, 18-months MACE in ACS patients. **(B)** *upper panel*, 18-months all-cause mortality in the STEMI, NSTEMI, and UAP subgroups; *lower panel*, 18-months MACE in the STEMI, NSTEMI, and UAP subgroups. *p < 0.05; **p < 0.001.

Subsequent multivariate Cox regression analysis adjusting for the traditional risk factors showed that a higher *Lactobacillus* level (>7.32 log_10_ copies/g) (aHR = 0.239; 95% CI: 0.093–0.617; p < 0.05) was significantly associated with a decreased risk of an all-cause death, while age above 65 years old (aHR = 1.922; 95% CI: 1.200–3.079; p < 0.05), history of MI (aHR = 5.392; 95% CI: 1.457–19.949; p < 0.05), and BNP level (aHR = 1.000; 95% CI: 1.000–1.000; p < 0.05) were significantly associated with an increased risk of an all-cause death (**Figure 3A**). A higher *Lactobacillus* level (>7.32 log_10_ copies/g) (aHR = 0.208; 95% CI: 0.081–0.531; p < 0.05) was significantly associated with a decreased risk of MACE, while age above 65 years old (aHR = 1.922; 95% CI: 1.133–3.373; p < 0.05), history of MI (aHR = 6.247; 95% CI: 1.585–24.000; p < 0.05), and UA (aHR = 1.005; 95% CI: 1.001-1.008; p<0.05) were significantly associated with an increased risk of MACE (**Figure 3A**). However, the type of ACS was not an independent factor for the prediction of all-cause death and MACE (all p > 0.05, **Figure 3A**). Furthermore, a higher *Lactobacillus* level was associated with a decreased risk of heart failure and revascularization (all p < 0.05, **Supplementary Table 4**).

After stratifying by the type of ACS (STEMI, NSTEMI, and UAP), the results of the univariate Cox regression analysis adjusted for the traditional risk factors exhibited that the association of *Lactobacillus* level with all-cause death and MACE was only observed in the STEMI subgroup but not in the NSTEMI and UAP subgroups (**Figure 3B**).

**Figure 4:**
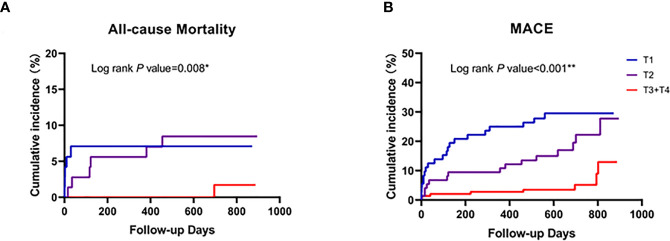
Comparison of the cardiovascular events post-PCI between patients with low and high *Lactobacillus* levels. Follow-up cardiovascular events included all-cause death **(A)** and the MACE-cumulative incidence **(B)**. Patients were stratified according to the high value of *Lactobacillus* level (T3+T4, 7.32 log_10_ copies/g) in ACS patients. *p < 0.05; **p < 0.001.

Kaplan-Meier curve analyses revealed that a median cutoff value of *Lactobacillus* level (>7.32 log_10_ copies/g) could identify the subjects at a higher risk for all-cause death or post-PCI MACE (**Figure 4**). In addition, lower *Lactobacillus* levels led to a more efficient risk stratification in ACS patients, which predicted the increase of MACE or all-cause death incidence rate (all p < 0.05, **Figure 4**).

## Discussion

The main finding of the present study is that the fecal *Lactobacillus* level is negatively associated with the SYNTAX score and more severe coronary artery lesions in patients with ACS. An additional major finding is the observed correlation between the *Lactobacillus* levels and evidence of myocardial necrosis (quantified by cTnT) in patients with ACS who underwent coronary angiography (Omland et al., 2009), although renal insufficiency could be a confounder. Furthermore, decreased *Lactobacillus* levels were an independent predictor of a higher SYNTAX score and elevated cTnT after adjustments for the traditional risk factors. The increase in the *Lactobacillus* level resulted in a significant increase in the AUC for the prediction of severe coronary atherosclerotic lesions, and *Lactobacillus* level was negatively associated with higher MACE and all-cause mortality rates after PCI. Taken together, the results of this study indicate that decreased *Lactobacillus* levels are associated with a higher risk of severe coronary atherosclerotic lesion and worse post-PCI prognosis among patients with ACS.

The present study demonstrated that the amount of *Escherichia coli*, Streptococcus, and *Enterobacteriaceae* was significantly higher than that of the control group, the number of *Lactobacillus* was significantly lower than that of the control group, and the number of *Helicobacter pylori* was similar between the groups, which was consistent with the results of our previous high-throughput study (Gao et al., 2020). This result indicates that there is a typical intestinal microbiota dysbiosis in ACS patients, which is consistent with previous studies (Li J. et al., 2017; Alhmouda et al., 2019). *Lactobacillus* is considered beneficial in the human gut, and play an important role in the health of the body such as the colonization resistance of intestinal pathogenic bacteria and the inhibition of the proliferation of endogenous potentially pathogenic bacteria (Lupp et al., 2007; Wu et al., 2012). Particularly, multivariate logistic regression analysis adjusting for the traditional risk factors suggested that *Lactobacillus* and *Escherichia coli* may be independent predictors of onset in ACS patients.

It is now recognized that the gut microbiota plays a crucial role in disease susceptibility, and that its metabolites are involved in host immune system regulation and induce the development of atherosclerosis (AS) and thrombotic diseases by mediating basal metabolic processes, such as host cholesterol metabolism, uric acid metabolism, oxidative stress, and inflammatory responses and thrombosis (Wang et al., 2011; Karlsson et al., 2012; Jandhyala et al., 2015; Korem et al., 2015; Yissachar et al., 2017). However, the relations between intestinal microbiota and the severity of lesions in ACS patients have not been clearly reported. Senthong et al. (2016a) studied the gut microbiota-derived metabolite TMAO and atherosclerotic plaque load of CAD patients, and reported that an increased TMAO level may aggravate the atherosclerotic plaque load. The current study showed that there was a significant negative correlation between fecal *Lactobacillus* levels and a medium-high SYNTAX score, suggesting that *Lactobacillus* may be a protective factor against plaque progression in ACS patients. In our previous study, we found that the serum TMAO level was significantly increased in ACS patients compared with thw healthy controls, and was negatively associated with the gut *Lactobacillus* level in ACS patients (Gao et al., 2020). According to these findings, we speculate that a lower *Lactobacillus* level increasing the ACS onset may be caused by a coronary atherosclerotic lesion that is related to TMAO.

A number of studies have indicated a relation between TMAO and incident MACE and mortality among patients with CAD (Senthong et al., 2016b; Fernandez-Ruiz, 2017; Li X.S. et al., 2017; Gao et al., 2020). The present study further showed that fecal *Lactobacillus* levels were negatively associated with all-cause mortality and MACE in ACS patients after PCI. Furthermore, the use of *Lactobacillus* levels led to a more efficient risk stratification in ACS patients with respect to the risk of MACE and all-cause mortality. Existing evidence indicates that the inflammatory response plays an important role in cardiac repair after an acute MI (Porrello et al., 2011; Aurora et al., 2014). The gut microbiota, on the other hand, is involved in regulating the host inflammatory response. Study has shown that after a MI, the induced inflammatory response can also cause cardiac remodeling and cardiac insufficiency while repairing the heart (Frangogiannis, 2012; Frangogiannis, 2014). In an acute MI, the intestinal barrier function is seriously impaired due to the weakening of the cardiac output and insufficient intestinal perfusion, which significantly increases the translocation of gut microbiota resulting in endotoxemia, a systemic inflammatory reaction, and multiple organ failure, all of which can further damage cardiomyocytes (Fujimura et al., 2010; Chen et al., 2011; Zhou et al., 2018). These changes increase the risk of cardiovascular events after a MI. However, these prior studies did not identify the specific gut microbes that are associated with the increased risk of cardiovascular events after a MI. The present study extended these observations, and suggested that lower *Lactobacillus* levels are related to an increased risk of MACE and all-cause death after PCI, and this may be due to the inflammatory reaction-mediated damage of cardiomyocytes.

Lam et al. (2012) showed that the administration of oral antibiotics or probiotics (containing *Lactobacillus plantarum* 299v and *Bifidobacterium* BI-07) before ischemia-reperfusion injury significantly reduced the infarct size and improved the myocardial function in rats, which demonstrated that the microbiota can affect ventricular remodeling after a MI. Tang et al. (2019) reported that antibiotic-treated mice (ABX mice) had a significantly increased mortality after a MI, and mortality was positively associated with the antibiotic dosage. However, after feeding the mice *Lactobacillus*, LVEF was improved after a MI, although there was no significant effect on the survival. The study confirmed that the gut microbiota plays an indispensable role in cardiac repair, especially in the initial stage after a MI.

TMAO has been shown to be associated with the pathogenesis of ACS and prognosis (Suzuki et al., 2017; Liu et al., 2018; Tan et al., 2019). Other studies have directly or indirectly demonstrated the involvement of the gut microbiota and metabolites in the development of ACS and its prognosis. Therefore, knowledge of gut microbiota changes in patients with ACS is essential for the prevention and treatment of the disease. Among the probiotic and pathogenic bacteria examined in the current study, *Lactobacillus* was shown to improve the prognosis of ACS patients, including reducing the all-cause death and MACE rates, as well as the rate of heart failure. This finding is consistent with that of Tang and colleagues (2019), and taken together, we can conclude that gut *Lactobacillus* may play an active role in the cardiac repair in patients with ACS, and even patients post MI.

Improving gut microecology has become a focus of cardiovascular research, and methods to improve the microecology include diet regulation, administration of antibiotics and probiotics, and fecal bacteria transplantation (Pallister and Spector, 2016; Smits et al., 2018). However, there is still a controversy about the use of antibiotics to prevent the occurrence of MACE after ACS. As mentioned earlier, Lam et al. and Tang et al. reported completely opposite results for animals treated with antibiotics after a MI (Lam et al., 2012; Tang et al., 2019). The cause of the contradictory results is not clear, but may be related to the bidirectional nature of the inflammatory response, and that a long-term use of antibiotics may also disrupt the normal gut microecological function.

There are limitations to the current study. There are up to 500 species with up to 10^14^ bacterial cells in the gut microbiota, and only 6 were examined in the current study. The effect of other bacteria on ACS has not been examined. The gut microbiota is involved in a variety of metabolic processes, including oxidative stress and the inflammatory response, and it may be expected that other bacteria may be involved in the regulation of these processes. We did not assess the effect of dietary factors on the SYNTAX score and prognosis in ACS patients. It is likely that severe ACS patients might have different dietary factors than patients in the control group or patients with milder forms of ACS.

## Conclusion

Fecal *Lactobacillus* level may be an important prognostic marker for predicting the severity of coronary artery lesion, clinical myonecrosis, and post-PCI MACE and all-cause death in patients with ACS. The prognostic effect is beyond that of the traditional risk markers. The significance of this finding is that the modification of the gut *Lactobacillus* level, with diet or new therapies underdevelopment, may play a role in the prevention and treatment of CAD and ACS.

## Data Availability Statement 

The original contributions presented in the study are included in the article/**Supplementary Material**. Further inquiries can be directed to the corresponding authors.

## Ethics Statement

The studies involving human participants were reviewed and approved by Tianjin Chest Hospital (No. 2018KY-010-01). The patients/participants provided their written informed consent to participate in this study.

## Author Contributions

JG had made substantial contributions to the conception. JG and YL contributed to the study design. JW, L-LZ, JM, and XZ preformed the experimental studies. JW, JM, XZ, JXW, and YW collected the data. T-TY, YC, JXW, YW, ZC, and YC analyzed the data. JG, JW, ZC, and YL prepared the manuscript. All authors contributed to the article and approved the submitted version.

## Funding

This research project was funded, in part, by the Key Project of Scientific and Technological Support Plan of Tianjin in 2020 (No.:20YFZCSY00820) and the Key Project of Healthcare Industry of Tianjin 2016 (No. 16KG131).

## Conflict of Interest

The authors declare that the research was conducted in the absence of any commercial or financial relationships that could be construed as a potential conflict of interest.

## Publisher’s Note

All claims expressed in this article are solely those of the authors and do not necessarily represent those of their affiliated organizations, or those of the publisher, the editors and the reviewers. Any product that may be evaluated in this article, or claim that may be made by its manufacturer, is not guaranteed or endorsed by the publisher.

## Abbreviations

ACS, acute coronary syndrome; CVD, cardiovascular disease; CAD, coronary artery disease; cTNT, cardiac troponin T; ESC/EACTS, European Society of Cardiology/European Association for Cardio-Thoracic Surgery; MACE, major adverse cardiac event; NSTEMI, non-ST-segment elevation myocardial infarction; PCI, percutaneous coronary intervention; STEMI, ST-segment elevation myocardial infarction; SYNTAX, SYNergy between percutaneous coronary intervention with (paclitaxel-eluting) TAXUS stent and cardiac surgery; TMAO, trimethylamine-N-oxide; UAP, unstable angina pectoris.
