# Supplementary material for: Gut Lactobacillus Level Is a Predictive Marker for Coronary Atherosclerotic Lesions Progress and Prognosis in Patients With Acute Coronary Syndrome
Source: Front Cell Infect Microbiol. 2021 Sep 7;11:687827. doi: 10.3389/fcimb.2021.687827 (PMC8452980; doi:10.3389/fcimb.2021.687827)
Supplement: Supplementary file 1 [file Table_1.docx]

**SUPPLEMENTAL MATERIAL**

**Supplementary Figure 1. Real-time PCR amplification curves of standard DNA fragments.**

**
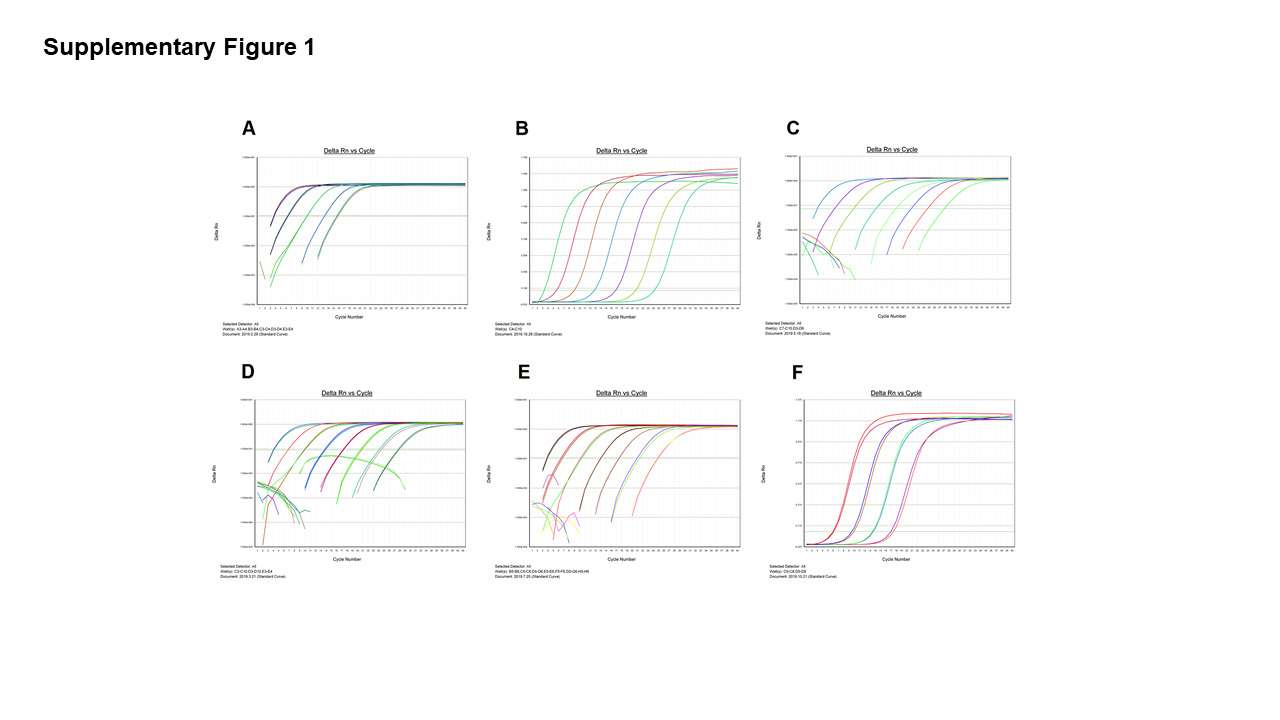
**

**Supplementary Figure 2. Standard curves of each bacterium.**

**
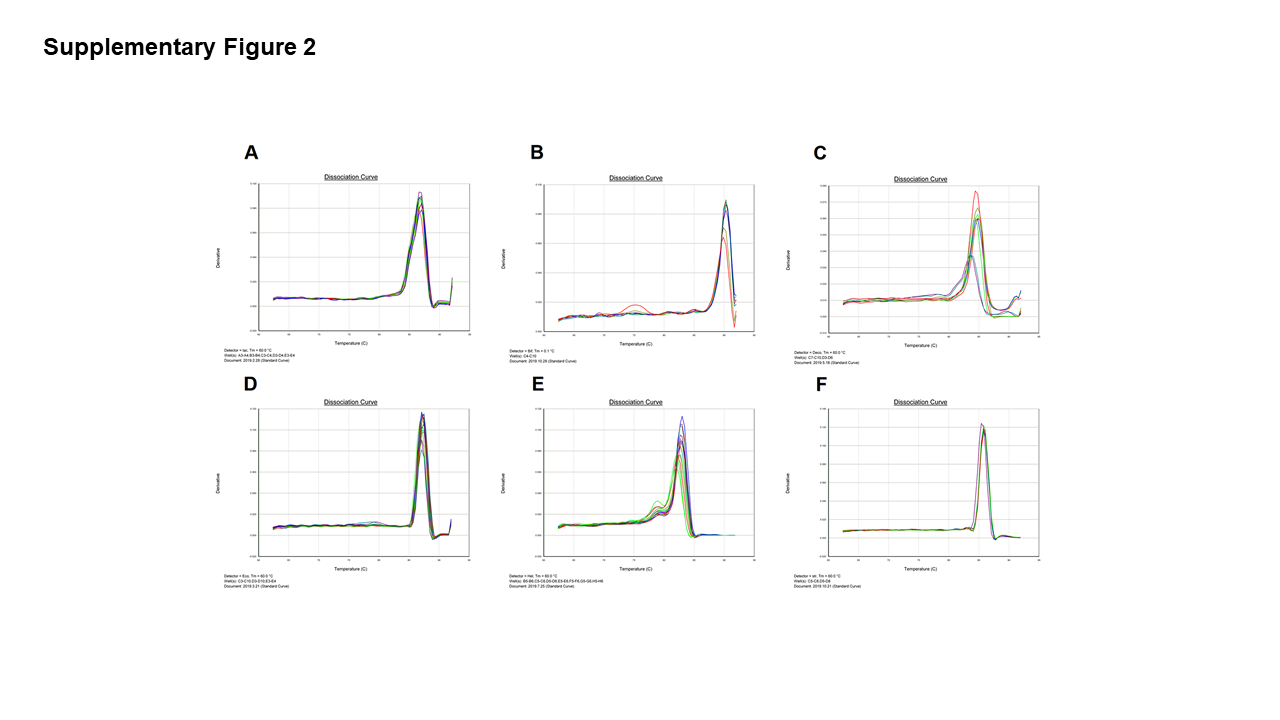
**

**Supplementary Figure 3. Dissociation curve analysis of amplified products.**

**
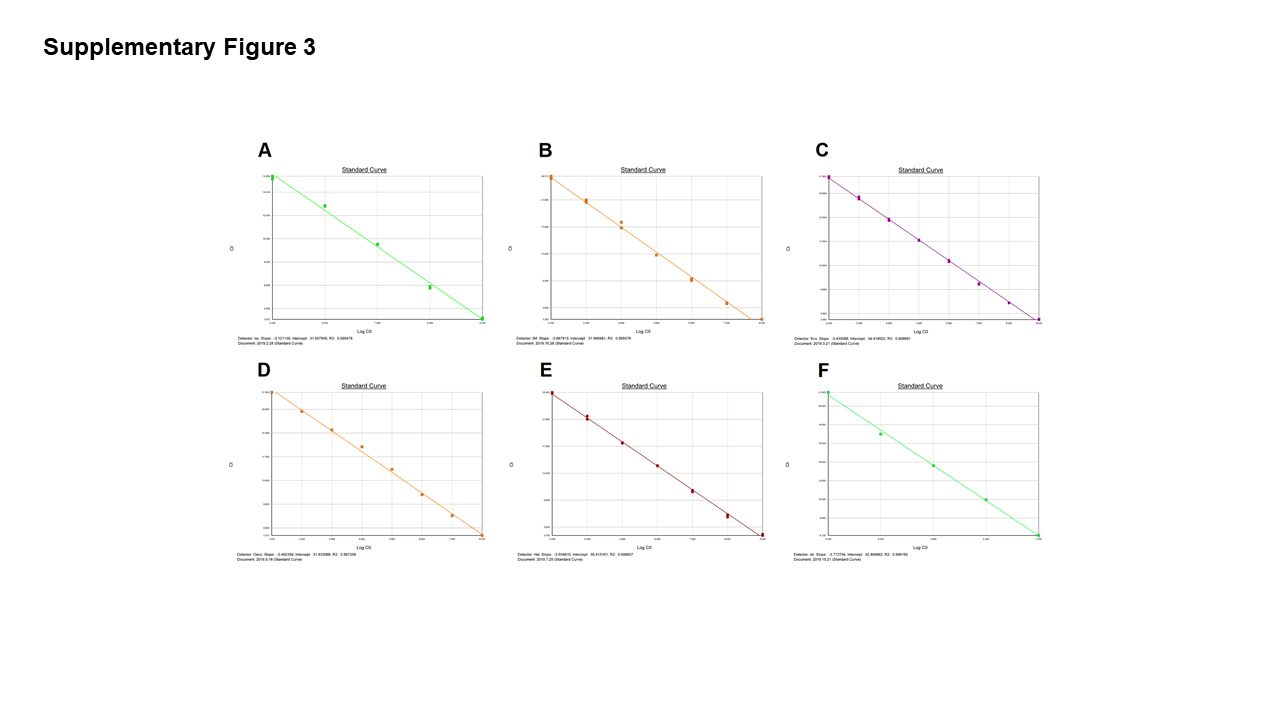
**

**Supplementary Figure 4. Receiver operator characteristic (ROC) curve analysis of Lactobacillus for identifying high SYNTAX score.**

**
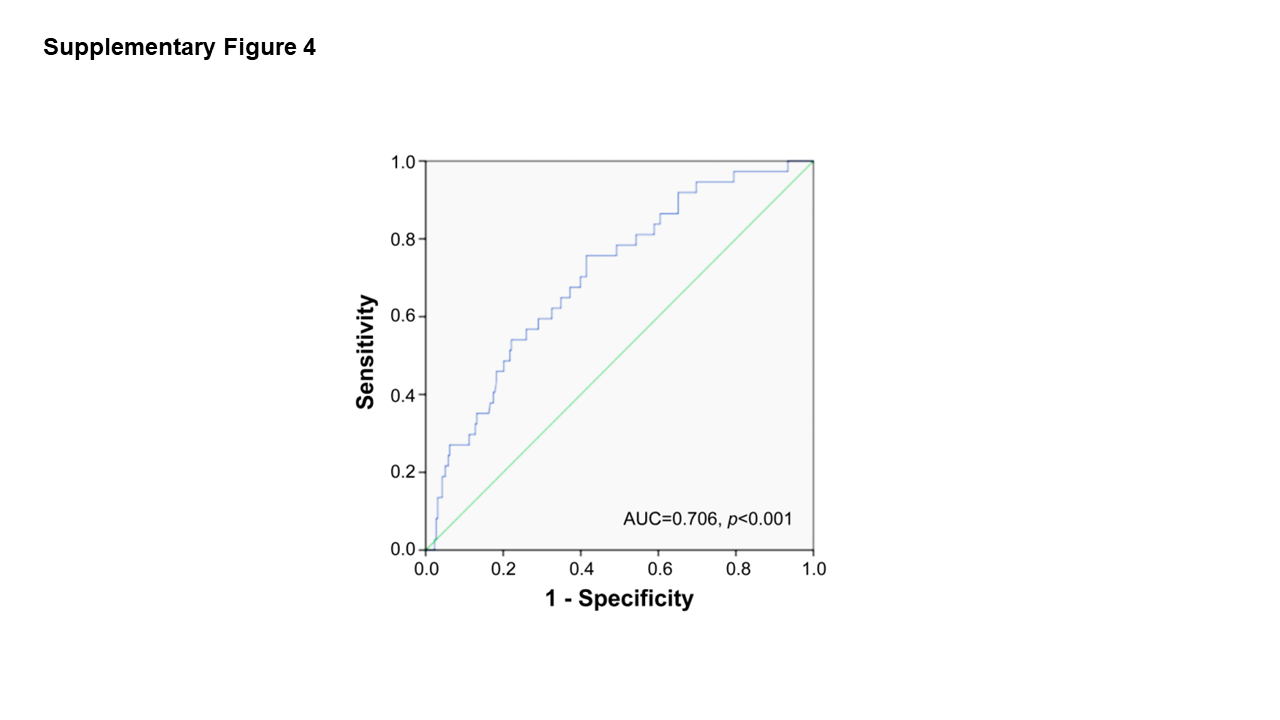
**

**Supplementary Figure legends**

**Supplementary Figure 1. Real-time PCR amplification curves of standard DNA fragments.** (A) *Lactobacillus*; (B) *Bifidobacteria*; (C) *Enterobacteriaceae*; (D) *Escherichia coli*; (E) *Helicobacter pylori*; (F) *Streptococcus*.

**Supplementary Figure 2. Standard curves of each bacterium.** (A) *Lactobacillus*; (B) *Bifidobacteria*; (C) *Enterobacteriaceae*; (D) *Escherichia coli*; (E) *Helicobacter pylori*; (F) *Streptococcus*.

**Supplementary Figure 3. Dissociation curve analysis of amplified products.** (A) *Lactobacillus*; (B) *Bifidobacteria*; (C) *Enterobacteriaceae*; (D) *Escherichia coli*; (E) *Helicobacter pylori*; (F) *Streptococcus*.

**Supplementary Figure 4. Receiver operator characteristic (ROC) curve analysis of Lactobacillus for identifying high SYNTAX score.** ROC analysis revealed that the diagnostic cutoff value of gut *Lactobacillus* level was 7.32 log_10_ copies/g, which yielded an area under the curve (AUC) of 0.706, sensitivity of 76%, and a specificity of 54%.

**Supplementary Table 1. Target bacteria and primer sequences**

| Bacterial species | **Primer** | **Sequences (5´-3´)** | **Annealing temperature (℃)** | **Amplification fragment size (bp)** |
| --- | --- | --- | --- | --- |
| *Bifidobacteria* | Bifid-F | CTCCTGGAAACGGGTGG | 55 | 550bp(Senthong et al., 2016) |
|  | Bifid-R | GGTGTTCTTCCCGATATCTACA |  |  |
| *Lactobacillus* | Lac-F | AGCAGTAGGGAATCTTCCA | 58 | 341bp(Ikeda et al., 2012;Tang et al., 2015) |
|  | Lac-R | CACCGCTACACATGGAG |  |  |
| *Escherichia coli* | Eco-F | CTCGCTGGCATTTGCGTAG | 58 | 347bp(Sianos et al., 2005) |
|  | Eco-R | ATCTTTTGCCGTCCGTTTTG |  |  |
| *Streptococcus* | Str-F | AGCTTAGAAGCAGCTATTCATTC | 60 | 309bp(Neumann et al., 2019) |
|  | Str-R | GGATACACCTTTCGGTCTCTC |  |  |
| *Helicobacter pylori* | Hel-F | GGAGCTGTCTCAACCAGAGATTC | 62 | 110bp(Mohr et al., 2013) |
|  | Hel-R | CGCATGATATTCCCATTAGCAG |  |  |
| *Enterobacteriaceae* | Eco-1457-F | CATTGACGTTACCCGCGAGAAGAAGC | 63 | 195bp(Serruys et al., 2009) |
|  | Eco-1652-R | CTCTACGAGCTCAAGCTTGC |  |  |

**Supplementary Table 2.** **Logistic regression analysis of patient characteristics associated with ACS occurrence**

|  | Univariate analysis | | Multivariate analysis | |
| --- | --- | --- | --- | --- |
|  | ***aOR (95% CI)*** | ***p-*value^a^** | ***aOR (95% CI)*** | ***p-*value^a^** |
| BMI (kg/m^2^) | 1.532(1.331,1.764) | <0.001* | 2.177(1.608,4.198) | 0.001* |
| Hypertension, n (%) | 2.731(1.419,5.255) | 0.003* |  |  |
| Diabetes, n (%) | 3.983(1.389,11.421) | 0.010* |  |  |
| LVEF (%) | 0.668(0.617,0.766) | <0.001* | 0.703(0.543,0.911) | 0.008* |
| Systolic pressure (mmHg) | 1.038(1.019,1.057) | <0.001* | 1.046(1.003,1.090) | 0.035* |
| TC (mmol/L) | 1.593(0.989,2.566) | 0.056 |  |  |
| HDL (mmol/L) | 0.080(0.032,0.202) | <0.001* |  |  |
| All bacteria (log_10_ copies/g) | 1.109(1.033,1.191) | 0.004* |  |  |
| *Lactobacillus* | 0.895(0.820,0.978) | 0.007* | 0.678(0.485,0.947) | 0.023* |
| *Escherichia coli* | 1.236(1.117,1.368) | <0.001* |  |  |
| *Streptococcus* | 1.100(0.996,1.215) | 0.060 |  |  |
| *Enterobacteriaceae* | 1.205(1.117,1.300) | <0.001* | 1.866(1.194,2.915) | 0.006* |

ACS, acute coronary syndrome; aOR, adjusted odds ratio; CI, confidence interval; BMI, body mass index; LVEF, Left ventricular ejection fraction; TC, total cholesterol; HDL, high density lipoprotein. ^a^Logistic regression analysis. **p*<0.05

**Supplementary Table 3. Association between *Lactobacillus* levels and cTNT in ACS patients**

| **Univariate analysis** | | | | | |
| --- | --- | --- | --- | --- | --- |
|  | **cTNT<0.6 (Low)** | **0.6≤cTNT≤9.7 (Medium)** | | **cTNT>9.7 (High)** | |
|  |  | ***aOR (95% CI)*** | ***p-*value^a^** | ***aOR (95% CI)*** | ***p-*value^a^** |
| ***Lactobacillus***(log_10_ copies/g) | Ref. | 0.905(0.853,0.961) | 0.001* | 0.848(0.758,0.949) | 0.004* |
| T1 (<4.21) | Ref. | Ref. |  | Ref. |  |
| T2 (4.21-7.32) | Ref. | 0.616(0.298,1.270) | 0.189 | 0.652(0.225,1.884) | 0.429 |
| T3+T4 (>7.32) | Ref. | 0.374(0.200,0.701) | 0.002* | 0.210(0.074,0.598) | 0.003* |
| **Multivariate analysis** | | | | | |
|  | **cTNT<0.6 (Low)** | **0.6≤cTNT≤9.7 (Medium)** | | **cTNT>9.7 (High)** | |
|  |  | ***aOR (95% CI)*** | ***p-*value** | ***aOR (95% CI)*** | ***p-*value** |
| ***Lactobacillus* (**log_10_ copies/g**)** | Ref. | 0.997(0.991,1.012) | 0.780 | 0.954(0.921,0.987) | <0.001* |
| T1 (<4.21) | Ref. | Ref. |  | Ref. |  |
| T2 (4.21-7.32) | Ref. | 0.551(0.247,1.229) | 0.146 | 0.683(0.208,2.238) | 0.529 |
| T3+T4 (>7.32) | Ref. | 0.462(0.233,0.915) | 0.027* | 0.317(0.099,0.914) | 0.042* |

ACS, acute coronary syndrome; cTNT max, cardiac troponin T max; aOR, adjusted odds ratio. ^a^Logistic regression analysis. **p*<0.05

**Supplementary Table 4. Association of *Lactobacillus* levels and prognosis in ACS patients**

|  | Univariate analysis | | Multivariate analysis | |
| --- | --- | --- | --- | --- |
|  | ***aHR (95% CI)*** | ***p-*value^a^** | ***aHR (95% CI)*** | ***p-*value^a^** |
| All-cause death | 0.860(0.748,0.989) | 0.034* | 0.854(0.735,0.907) | 0.038* |
| MACE | 0.812(0.740,0.892) | <0.001* | 0.828(0.742,0.922) | <0.001* |
| Cardiac death | 0.751(0.618,0.912) | 0.004* | 0.758(0.573,1.004) | 0.053 |
| Recurrent AMI | 0.748(0.556,1.006) | 0.055 | - | - |
| Revascularization | 0.554(0.330,0.930) | 0.025* | 0.441(0.208,0.934) | 0.033* |
| Heart failure | 0.786(0.661,0.935) | 0.007* | 0.768(0.620,0.950) | 0.015* |
| Stroke | 0.961(0.790,1.168) | 0.688 | - | - |
| Angina | 0.967(0.853,1.096) | 0.598 | - | - |

ACS, acute coronary syndrome; MACE, major adverse cardiovascular event; aHR, adjusted hazard ratio; CI, confidence interval. ^a^Cox regression analysis. **p*<0.05

**References**

Ikeda, N., Kogame, N., Iijima, R., Nakamura, M., and Sugi, K. (2012). Carotid artery intima-media thickness and plaque score can predict the SYNTAX score. *Eur Heart J* 33**,** 113-119.

Mohr, F.W., Morice, M.C., Kappetein, A.P., Feldman, T.E., Stahle, E., Colombo, A., Mack, M.J., Holmes, D.R., Jr., Morel, M.A., Van Dyck, N., Houle, V.M., Dawkins, K.D., and Serruys, P.W. (2013). Coronary artery bypass graft surgery versus percutaneous coronary intervention in patients with three-vessel disease and left main coronary disease: 5-year follow-up of the randomised, clinical SYNTAX trial. *Lancet* 381**,** 629-638.

Neumann, F.J., Sousa-Uva, M., Ahlsson, A., Alfonso, F., Banning, A.P., Benedetto, U., Byrne, R.A., Collet, J.P., Falk, V., Head, S.J., Juni, P., Kastrati, A., Koller, A., Kristensen, S.D., Niebauer, J., Richter, D.J., Seferovic, P.M., Sibbing, D., Stefanini, G.G., Windecker, S., Yadav, R., Zembala, M.O., and Group, E.S.C.S.D. (2019). 2018 ESC/EACTS Guidelines on myocardial revascularization. *Eur Heart J* 40**,** 87-165.

Senthong, V., Wang, Z., Li, X.S., Fan, Y., Wu, Y., Tang, W.H., and Hazen, S.L. (2016). Intestinal Microbiota-Generated Metabolite Trimethylamine-N-Oxide and 5-Year Mortality Risk in Stable Coronary Artery Disease: The Contributory Role of Intestinal Microbiota in a COURAGE-Like Patient Cohort. *J Am Heart Assoc* 5.

Serruys, P.W., Morice, M.C., Kappetein, A.P., Colombo, A., Holmes, D.R., Mack, M.J., Stahle, E., Feldman, T.E., Van Den Brand, M., Bass, E.J., Van Dyck, N., Leadley, K., Dawkins, K.D., Mohr, F.W., and Investigators, S. (2009). Percutaneous coronary intervention versus coronary-artery bypass grafting for severe coronary artery disease. *N Engl J Med* 360**,** 961-972.

Sianos, G., Morel, M.A., Kappetein, A.P., Morice, M.C., Colombo, A., Dawkins, K., Van Den Brand, M., Van Dyck, N., Russell, M.E., Mohr, F.W., and Serruys, P.W. (2005). The SYNTAX Score: an angiographic tool grading the complexity of coronary artery disease. *EuroIntervention* 1**,** 219-227.

Tang, W.H., Wang, Z., Shrestha, K., Borowski, A.G., Wu, Y., Troughton, R.W., Klein, A.L., and Hazen, S.L. (2015). Intestinal microbiota-dependent phosphatidylcholine metabolites, diastolic dysfunction, and adverse clinical outcomes in chronic systolic heart failure. *J Card Fail* 21**,** 91-96.
